# Performance of HPV16/18 in Triage of Cytological Atypical Squamous Cells of Undetermined Significance

**DOI:** 10.1155/2019/4324710

**Published:** 2019-12-16

**Authors:** Xiaoqin Cao, Shuzheng Liu, Manman Jia, Hongmin Chen, Dongmei Zhao, Bing Dong, Zhen Guo, Lingyan Ren, Shaokai Zhang, Xibin Sun

**Affiliations:** ^1^Department of Cancer Epidemiology, Affiliated Cancer Hospital of Zhengzhou University, Henan Cancer Hospital, Zhengzhou 450008, China; ^2^Gynecologic Oncology Department, Affiliated Cancer Hospital of Zhengzhou University, Henan Cancer Hospital, Zhengzhou, Henan 450008, China; ^3^Pathology Department, Affiliated Cancer Hospital of Zhengzhou University, Henan Cancer Hospital, Zhengzhou, Henan 450008, China; ^4^Molecular Pathology Department, Affiliated Cancer Hospital of Zhengzhou University, Henan Cancer Hospital, Zhengzhou, Henan 450008, China; ^5^Central Laboratory, Affiliated Cancer Hospital of Zhengzhou University, Henan Cancer Hospital, Zhengzhou, Henan 450008, China

## Abstract

**Context:**

Human papillomavirus (HPV) testing is widely used in cervical cancer screening in women; however, its efficiency in triaging women with atypical squamous cells of undetermined significance (ASC-US) needs to be validated.

**Objective:**

To evaluate the performance of HPV16/18 in the triage of women with ASC-US.

**Methods:**

Women presenting for routine cervical cancer screening had cervical specimens collected, with which both liquid-based cytology (LBC) and hrHPVs were examined; those with ASC-US cytology underwent colposcopy. HPV16/18 and 12 other types were tested with domestic hybridization capture and chemiluminescence signal amplification (DH3). Performance characteristics of HPV test (sensitivity, specificity, positive predictive value (PPV), negative predictive value (NPV) for identification of cervical intraepithelium neoplasma (CIN) grade 2 or worse (CIN2+), and CIN grade 3 or worse (CIN3+)) were determined using standard statistical tests.

**Results:**

317 women with ASC-US were eligible for the study. HrHPV prevalence was 15.77% (50/317); HPV16/18 prevalence was 3.61% (20/317). Sensitivity and specificity of HPV16/18 for detection of CIN 2+ were 64.71% and 97% and 64.29% and 96.37% for detection of CIN 3+, respectively. The positive predictive values (PPVs) and negative predictive values (NPVs) of HPV16/18 were 55.00% and 97.98% for CIN2+ and 45.00% and 98.32% for CIN3+, respectively.

**Conclusion:**

HPV16/18 can be considered as an effective method to triage women with ASC-US as its good clinical performance.

**Trial Registration:**

This trial is registered with Henan Cancer Hospital Medical Ethics Committee on July 5, 2016 (http://www.anti-cancer.com.cn), with registry no.: 2016037.

## 1. Background

Cervical cancer screening methodology has evolved and benefited from detection of high-risk human papillomavirus (hrHPV) as HPV infection has been verified as the leading carcinogenesis of cervical cancer [1, 2]. Atypical squamous cells of undetermined significance (ASC-US) is an important precursor lesion process [3]. In the clinics, how to refer women with ASC-US for further diagnosis has a dispute, for a repeatable cytological examination, HPV test, or further colonoscopy.

As it is known, hrHPV DNA genotypes were generally categorized as 16, 18, 31, 33, 35, 39, 45, 51, 52, 56, 58, 59, 66, and 68, among which HPV16 and HPV18 were generally recognized as the highest risk of cervical cancer genesis [4–7]. In 2016, the Society of Gynecologic Oncology (SGO) released that either HPV16- or HPV18-positive women should be referred directly for colposcopy [8]. Several studies have shown that, compared to repeated cytology, HPV DNA assays have a good performance in finding high-grade CIN, and a negative HPV DNA test result has a very high negative predictive value [9, 10]. However, there were few studies that assessed the performance of HPV16/18 in the triage of ASC-US. Meanwhile, hrHPV prevalence varied in different countries and regions. It lacks efficient evidences on the triage of women with cytological ASC-US for colposcopy or repeat cytology in China.

In this study, we aimed to assess the performance of HPV16/18 on the triage of women with ASC-US cytology in a central Chinese population.

## 2. Methods

### 2.1. Study Population and Protocol

This study is based on a field epidemiology clinical test. The women were recruited with group sampling method from Xinmi city, China.

The included criteria were as follows: age between 21 and 64 years, intact uterus, with cytological ASC-US, willing and able to undergo colposcopy, and agreeing to participate within 12 weeks. Exclusion criteria included pregnancy or 8 weeks postpartum, history of cervix surgery or pelvic radiation therapy, cervical cancer, or precancerous lesions. The first participant of the trial started on Nov. 8, 2016. All the participants signed the informed consents.

All the participants were routinely collected cervical exfoliative cell specimen. The ThinPrep cytological test (TCT) was a canonical screening method for cervical cancer. HrHPV detection employed the principle of domestic hybridization capture and chemiluminescence signal amplification (DH3), a qualitative method to determine HPV16/18 genotypes and the other 12 types of hrHPV but HPV16/18 (HPV31, 33, 35, 39, 45, 51, 52, 56, 58, 59, 66, 68, and HPV-O-12). HPV specimen preservation solution was provided by Hangzhou De Tong Biotechnology Co., Ltd, China. Women with cytological ASC-US or worse were referred for colposcopy and necessary pathological test. All the processes were put under quality control system.

### 2.2. Statistical Analysis

Data were collected using Microsoft Access 2013 software by two recorders. Microsoft Visual FoxPro 9.0 was used for checking. Performances were estimated through OpenEpi, version 3, by sensitivity, specificity, positive predictive value (PPV), and negative predictive value (NPV).

## 3. Results

### 3.1. Characteristics of Women with ASC-US

A total of 3050 women received cytological examination and HPV detection. 2624 (86.03%) women were cytologically normal and 426 (13.97%) were above ASC-US (ASC-US: 335, LSIL: 87, ASC-H: 1, and HSIL: 3). In the end, 317 women with ASC-US completed the triaging program. The general information process is listed in Table 1.

### 3.2. HPV Distribution in ASC-US Population with Histological Diagnoses

Performance of different groups of hrHPV16/18 or HPV-O-12 is listed and sorted (Table 2). HPV prevalence was 15.77% (50/317) and HPV16/18 prevalence was 3.61% (20/317).

### 3.3. HrHPV Performance in Triaging ASC-US Population

Using histologically confirmed CIN2+ and CIN3+ lesions as the gold standard, we estimated the performance for the DH3 HPV assay (Tables 3 and 4, respectively). The positive predictive values (PPVs) and negative predictive values (NPVs) of hrHPVs for CIN2+ were 28.00% and 98.88% and for CIN3+, 24.00% and 99.25%, respectively. As for HPV16/18, PPVs and NPVs were 55.00% and 97.98% for CIN2+ and 45.00% and 98.32% for CIN3+, respectively.

## 4. Discussion

Cytology is still widely used in cervical cancer screening, and ASC-US triage remains ambiguous. Some research reported that ASC-US accounted for 5%~10% of cervical cancer screening population. In this study, we got 335 ASC-US from 3015 women (11.11%). CIN2+ and CIN3+ detective rates were 5.36% (17/317) and 4.42 (14/317) of ASC-US population. They were similar with other studies. Pan et al. [11] reported a CIN2+ detection rate of 3.2% from ASC-US and 15.3% from LSIL in a pooled analysis in China. However, cumulative time and participants in clinic or hospital increased the risk ratio greatly. ALTS group reported a 2-year cumulative diagnosis of CIN grade 3 was 8% to 9% [12]. Studies showed a wide range of detective ratio, from 8% to 31.71%, with women from clinics or inpatients [13, 14].

In our study, hrHPV prevalence rate was 15.77% in women with ASC-US and HPV16/18 infection rate was 6.31%. Different genotypes of HPV prevalence varied in cervical cytological status and in different populations. In some European countries, the higher positive rate of type is HPV16, 31, and 51 [15, 16]. It was reported that the most prevalent types were HPV16, 18 and 52 in inner Mongolia, China [17]. Another cohort study in Shanxi, China, found that during the 10-year follow-up, the infection rate of HPV16 decreased, while HPV52 increased [18]. These suggested HPV prevalence in different regions or populations, which would lead to associated strength width between HPV infection and cervical cancer incidence.

Performances of HPV show that HPV16/18 has an optimal specificity; it was 97.00% for CIN2+ and 96.37% for CIN3+. HrHPVs had a superior sensitivity and NPV compared to HPV16/18. In general, HPV16/18 performed well for ASC-US triage for its high diagnostic accuracy, which were both above 95% for CIN3+ and CIN2+. When we expect a higher sensitivity, we recommend to discriminate HPV genotypes in ASC-US population, since HPV subtypes showed different carcinogenic potential in many studies. Wong et al. [19] stratified the absolute risk for progression in women with ASC-US, based upon hrHPV genotype detection at the baseline screening. They concluded that HPV16 had the highest risk for CIN grade 3 progression in women with ASC-US, which was fivefold greater than the collective risk attributable to infections with other hrHPV types. Another study in America reported that in addition to HPV16 and HPV18, 31, 33, 35, 39, 45, 51, 52, 56, 58, 59, 68, 73, and 82 should be considered carcinogenic, or high-risk types, and types 26, 53, and 66 should be considered probably carcinogenic [20]. Some proved HPV16, 18, and 45 counted for 77% of hrHPVs [20, 21]. de Sanjose et al. suggested that type-specific high-risk HPV DNA-based screening tests and protocols should focus on HPV types 16, 18, and 45 [5]. In China, Guan et al. reported the 6 most prevalent HPVs: HPV16, 33, 58, 56, 18, and 31 [22]. Ran [23] found that the most common types were HPV52, 58, and 33 in addition to types 16 and 18. All the above indicated multiple hrHPV prevalences and potential carcinogenic HPV genotypes. It seemed necessary to clarify the most high-risk HPV genotypes and furthermore to explore more optimal triage strategy. Further studies should focus on high-risk HPV genotypes, to select more major influential hrHPV genotypes besides HPV16/18, hence to get an optimal joint HPV group and make it feasible for clinical manipulation.

To sum up, HPV16/18 can be considered as an alternative method to current cytology-based ASC-US triage methods because of its high accuracy; however, predictive performance might be augmented to explore more potential carcinogenic HPV DNA genotypes in addition to 16/18.

## Figures and Tables

**Table 1:** Characteristics of the women with ASC-US cytology (*n* = 317).

Categorical variables	Frequency (%)	Continuous variables	Mean (min, max)
Educational level		Age	51.44 (23, 64)
Elementary school and below	164 (51.8)	Menstrual onset (yr)	15.45 (11, 20)
Middle school and higher	153 (48.2)	First pregnancy (yr)	23.06 (18, 37)
Smoking (never)	317 (100)	First delivery (yr)	23.55 (18, 38)
Drinking (never)	311 (98.11)	Pregnancy times	3.15 (0, 11)
Normal marriage status	303 (95.6)	Delivery times	2.4 (1 ,6)
Condom usage	8 (2.5)	Live birth times	2.37 (1, 6)

**Table 2:** HPV distribution with histological diagnoses in women with ASC-US (*n* = 317). Distribution of HPV in histological diagnoses (%).

HPV genotype	Total	Normal (*N* = 291)	CIN1 (*N* = 9)	CIN2 (*N* = 3)	CIN3 (*N* = 13)	CC (*N* = 1)
HPV16/18 (+)	20 (3.61)	9 (2.84)	0 (0)	2 (0.63)	8 (2.52)	1 (0.32)
HPV-O-12 (+)^∗^	39 (12.30)	29 (9.15)	1 (0.32)	1 (0.32)	8 (2.52)	0 (0)
HPVs (+)^∗^	50 (15.77)	35 (11.04)	1 (0.32)	2 (0.63)	11 (3.47)	1 (0.32)
HPV (-)	267 (84.23)	256 (80.76)	8 (2.52)	1 (0.32)	2 (0.63)	0 (0)

Note: ^∗^HPV indicated 14 HPV genotypes: 16, 18, 31, 33, 35, 39, 45, 51, 52, 56, 58, 59, 66, and 68; HPV-O-12 included 12 HPV genotypes: 31, 33, 35, 39, 45, 51, 52, 56, 58, 59, 66, and 68.

**Table 3:** HPV distribution with histological diagnoses in women with ASC-US (*n* = 317).

	CIN2+ (*n* = 17)	<CIN2 (*n* = 300)	CIN3+ (*n* = 14)	<CIN3 (*n* = 303)
HPV16/18 (+)	11	9	9	9
HPV16/18 (-)	6	291	5	294
HrHPV (+)	14	36	12	38
HrHPV (-)	3	264	2	265
HPV-O-12 (+)	9	30	8	31
HPV-O-12 (-)	8	270	6	272

**Table 4:** Performance of HPV tests for ASC-US triage.

Test type	Sensitivity	Specificity	PPV	NPV	Diagnostic accuracy	LR of a positive test	LR of a negative test
HrHPV for CIN3+	85.71 (60.06, 95.99)	87.46 (83.25, 90.73)	24.00 (14.3, 37.41)	99.25 (97.31, 99.79)	87.38 (83.27, 90.6)	6.84 (6.32-7.40)	0.16 (0.061-0.44)
HrHPV-O-12 for CIN3+	57.14 (32.59, 78.62)	89.77 (85.84, 92.7)	20.51 (10.78, 35.53)	97.84 (95.37, 99.01)	88.33 (84.33, 91.41)	5.59 (4.36-7.15)	0.48 (0.34-0.66)
HPV16/18 for CIN3+	64.29 (38.76, 83.66)	97.03 (94.45, 98.43)	50.00 (29.03, 70.97)	98.33 (96.15, 99.28)	95.58 (92.72, 97.35)	21.64 (15.42-30.37)	0.37 (0.25-0.54)
HrHPV for CIN2+	82.35 (58.97, 93.81)	88.00 (83.83, 91.2)	28.00 (17.47, 41.67)	98.88 (96.75, 99.62)	87.7 (83.62, 90.87)	6.86 (6.31-7.47)	0.20 (0.10-0.39)
HPV-O-12 for CIN2+	52.94 (30.96, 73.84)	90.00 (86.08, 92.91)	23.08 (12.65, 38.34)	97.12 (94.43, 98.53)	88.01 (83.97, 91.14)	5.29 (4.09-6.86)	0.52 (0.41-0.67)
HPV16/18 for CIN2+	64.71 (41.3, 82.69)	97.00 (94.4, 98.41)	55.00 (34.21, 74.18)	97.98 (95.66, 99.07)	95.27 (92.34, 97.11)	21.57 (15.74-29.55)	0.36 (0.26-0.50)

PPV: positive predictive value; NPV: negative predictive value; LR: likelihood ratio.

## Data Availability

No data were used to support this study.

## Abbreviations

HPV: Human papilloma virus
HrHPV: High-risk human papilloma virus
CIN: Cervical intraepithelial neoplasia
ASC-US: Atypical squamous cells of undetermined significance
SGO: Society of Gynecologic Oncology
ECC: Endocervical curettage
PPV: Positive predictive value
NPV: Negative predictive value.
